# Increase in plasma succinate is associated with aerobic lactate production in a model of endotoxic shock

**DOI:** 10.1113/EP092109

**Published:** 2025-03-19

**Authors:** Juan D. Caicedo Ruiz, Jorge I. Alvarado Sanchez, Juan J. Diaztagle Fernández, Cándida Diaz Brochero, Luis E. Cruz Martinez

**Affiliations:** ^1^ Department of Intensive Care Fundación Santa Fe de Bogotá Bogotá Colombia; ^2^ Physiology Division Department of Physiological Sciences Faculty of Medicine Universidad Nacional de Colombia Bogotá Colombia; ^3^ Department of Intensive Care Medicine Fundación Valle del Lili Cali Colombia; ^4^ Fundación Universitaria de Ciencias de la Salud, Department of Internal Medicine Hospital de San José Bogotá Colombia; ^5^ Pontificia Universidad Javeriana, Department of Internal Medicine, Hospital Universitario San Ignacio Bogotá Colombia

**Keywords:** endotoxic shock, Krebs cycle, lactate, sepsis, succinate, translational research

## Abstract

The Krebs or tricarboxylic acid (TCA) cycle plays a key role in the regulation of immune responses and adaptations to hypoxia that occur during sepsis. Although the concentrations of some of these intermediates have been reported to be increased in large cohorts of septic patients, a detailed analysis of their changes during sepsis is still lacking. Here, we investigated the plasma concentrations of several TCA intermediates in a swine model of endotoxic shock and the relationship between these TCA cycle intermediates and lactate production. Nine female swine were administered lipopolysaccharide to induce endotoxic shock, while four females served as controls. Plasma samples were collected at three time points: baseline, 3 and 6 h after lipopolysaccharide administration. Control samples were collected at parallel time points. Quantification of TCA intermediates, lactate and pyruvate was performed by high‐performance liquid chromatography. Oxygen‐derived variables were obtained by gas analysis of arterial and venous samples. The endotoxic shock group showed a significant increase in lactate, accompanied by stability of oxygen‐derived variables and a low lactate:pyruvate ratio, indicative of aerobic conditions. Of all the TCA intermediates analysed, only citrate and succinate showed significant increases compared with controls. Furthermore, the changes in lactate were determined, in part, by the changes in succinate concentration. The increase in succinate concentrations was associated with the increase in lactate in global aerobic conditions. Our results suggest a potential role for succinate as a biomarker of aerobic lactate production.

## Abstract (highlights)

- **What is the central question of this study?**
  Krebs cycle intermediates are associated with the metabolic reprogramming that occurs during sepsis. However, the temporal behaviour of these metabolites during endotoxaemia has not been documented previously.
- **What is the main finding and its importance?**
  Our study details the plasma concentrations of several Krebs cycle intermediates during endotoxic shock; such description has not been reported previously. Our findings suggest that changes in succinate concentrations are associated with variations in lactate concentrations during the early phase of endotoxic shock. Our results suggest a potential role for succinate as a biomarker of aerobic lactate production.

## 1 INTRODUCTION

The mechanisms that explain metabolic adaptation in septic shock have been studied extensively (Gómez et al., 2017; Weis et al., 2017). Recently, the incorporation of metabolomics in animal research models and critically ill patients has improved the understanding of complex metabolic adaptations that take place during sepsis, allowing the identification of different metabolites that accumulate in different phases of the disease (Izquierdo‐Garcia et al., 2019; Lado‐Abeal et al., 2018; Langley et al., 2014; Whelan et al., 2014). Among these metabolites are the intermediates of the Krebs cycle or tricarboxylic acid (TCA) cycle, which are associated with the metabolic reprogramming that occurs during states of hypoxia, ischaemia and inflammation (Chouchani et al., 2014; Hussain et al., 2022; Koivunen et al., 2008; Martínez‐Reyes & Chandel, 2020; Nunns et al., 2016).

Certain TCA cycle intermediates have received particular attention in research. Succinate, for instance, has been found to be increased in animal models of haemorrhagic shock (D'Alessandro et al., 2015; Reisz et al., 2018) and in patients with hypovolaemic traumatic shock, in whom a persistent increase in succinate was linked to higher mortality rates (D'Alessandro et al., 2017). Citrate has been related to the production of reactive oxygen species, nitric oxide and inflammatory mediators in lipopolysaccharide (LPS)‐activated immune cells (Infantino et al., 2011, 2014; Williams & O'Neill, 2018). Additionally, there is evidence that these and other TCA cycle intermediates, such as oxaloacetate and fumarate, increase the stabilization of hypoxia‐induced factor‐1α, leading to adaptation to hypoxia (Koivunen et al., 2017; Lukyanova et al., 2018; Selak et al., 2005).

Tricarboxylic acid cycle intermediates in patients with sepsis have been reported to show variable plasma concentrations; some studies have found increased concentrations of TCA intermediates in non‐surviving patients (Langley et al., 2014; Liu et al., 2016; Mickiewicz et al., 2014), contrasting with evidence of increased concentrations in patients with less severe infectious complications (Fedotcheva et al., 2015). The above results indicate that there are different metabolic profiles during the course of sepsis and that plasma concentrations of TCA intermediates are likely to be time dependent. In the present study, we develop an endotoxic shock model in swine to elucidate the changes in TCA intermediates. Although various studies of endotoxic shock have been performed in mice and rats (Hernandez‐Baixauli et al., 2023; Wang et al., 2020), swine have immunological and metabolic responses displaying a close resemblance to humans (Vintrych et al., 2023), therefore justifying the performance of experimental studies in this higher species. The purpose of this investigation was to identify the course of plasma TCA cycle intermediates in a swine model of endotoxic shock and to analyse its relationship with dynamic changes in lactate concentration and the lactate:pyruvate (L/P) ratio as a marker of anaerobiosis.

## 2 MATERIALS AND METHODS

All methods in this research were conducted according to the Animal Research: Reporting of In Vivo Experiments (ARRIVE) guidelines (Supplemental digital content 1) (Kilkenny et al., 2010). The study was developed according to the recommendations of resolution 008430 of 1993 of the Colombian Ministry of Health, Law 84 of 1989, Statute for the Protection of Animals, and the principles for the care and use of animals in research established by the *Guide for the Care and Use of Laboratory Animals* (US National Institutes of Health guide) (National Research Council, 2011).

### 2.1 Animal preparation

The study received ethical approval from the Bioethics Committee of the Faculty of Veterinary Medicine at Universidad Nacional de Colombia (CB‐FMVZ‐UN‐013‐17). It was carried out between January 2020 and December 2022 at the simulation laboratory of the Instituto de Simulación Médica (INSIMED) in Bogotá, Colombia. The process of animal preparation has been detailed in previously published studies by our group (Alvarado Sánchez et al., 2023, 2022). Briefly, 13 female Yorkshire pigs weighing ∼40 kg, ∼5 months of age, were farmed in an out‐of‐town bioterium facility. Animals were fasted overnight prior to the experiment with ad libitum access to water. The animals were premedicated with tiletamine–zolazepam used at a dose of 4.4 mg/kg intramuscularly. Later, the pigs were cannulated in the marginal vein of the ear, and induction was performed with isoflurane by face mask at 1.5% to perform orotracheal intubation with a 6.5 Fr endotracheal tube. For the maintenance of general anaesthesia, isoflurane at a dose of 1.5 minimum alveolar concentration (MAC) was used.  Intravenous fentanyl at a dose of 0.03–0.05 µg/kg/min was used as concomitant intravenous anaesthesia to avoid pain in the animals. Fluid infusion of normal saline solution was administered throughout the experiment at an infusion rate of 3 mL/kg/h.

### 2.2 General monitoring

Once it was determined that the swine had an adequate depth of anaesthesia, a central venous catheter was inserted into the right internal jugular vein for sampling, infusion of solutions and quantification of central venous pressure. Additionally, a femoral artery catheter coupled to a thermistor was inserted for arterial pulse contour analysis. The catheters were inserted by anatomical dissection, and their positioning was confirmed by their respective pressure curves. For central venous catheters, the presence of a central venous pressure wave with a and v waves and respiratory oscillations was indicative of correct placement, and for femoral artery catheters, the presence of arterial pressure wave was sufficient to confirm correct position. Quantification of cardiac output and global end‐diastolic volume was performed by transpulmonary thermodilution using a PiCCO system (PULSION Medical Systems AG, Munich, Germany) system, and systemic vascular resistance (SVR) was obtained by the formula SVR = mean arterial pressure/cardiac output. Furthermore, the body temperature of the swine was maintained carefully between 37°C and 38°C.

### 2.3 Experimental protocol

**FIGURE 1:**
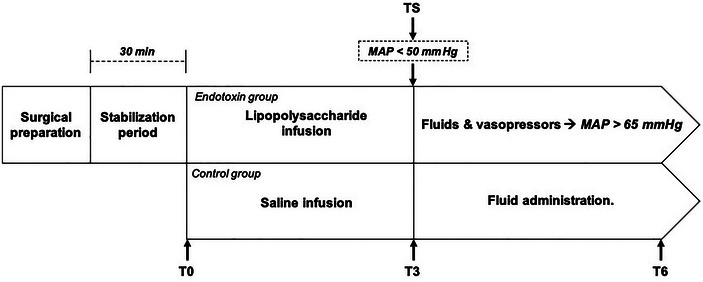
Experimental time line for endotoxin and control groups. Surgical preparation (sedation, muscular paralysis, endotracheal intubation, catheter insertion: subclavian vein, femoral artery‐transpulmonary thermodilution system). Stabilization period lasted ≥30 min. Blood samples were obtained in both groups at 0, 3 and 6 h (T0, T3 and T6, respectively). After T6, the experiment was stopped in both groups. Abbreviation: MAP, mean arterial pressure; TS, time of shock.

The experimental time line is summarized in Figure 1. The stabilization period was defined by a mean arterial pressure (MAP) variability of <10%; after this, baseline (T0) samples were taken. Then, the animals were divided into two groups: a control group (*n* = 4) and an endotoxin group (*n* = 9). In the endotoxin group, infusion of LPS from *Escherichia coli* 055:B5 (Sigma, St. Louis, MO, USA) was started at 7 µg/kg/min and escalated after 10 min to 14 µg/kg/min and to 20 µg/kg/min after 10 min; this infusion protocol has been used consistently by our group and others (Hatib et al., 2011) to induce endotoxic shock in pigs. The infusion of endotoxin was terminated when MAP fell below 50 mmHg for a minimum of 10 min; at this point, time of shock (TS) was declared. After TS in the endotoxin group, a fluid load was administered at a dose of 20 mL/kg for 20–30 min followed by noradrenaline infusion, starting at 0.05 µg/kg/min and increased by 0.05 µg/kg/min every 5 min until a MAP of 65 mmHg was reached. In the control group, endotoxin was not administered. A similar fluid load was administered at 3 h of observation. Timing in the control group was referenced from data acquired in the standardization phase, matching the TS in the endotoxin group with a T3 reference point in the control pigs. Noradrenaline was not administered to control animals.

Arterial and venous blood samples were obtained in both groups at T0, 3 h later (T3) and 6 h later (T6) for blood gas analysis and for quantification of TCA cycle intermediates. One millilitre of arterial and venous blood was analysed using an AVL OMNI™ 1–9 (Radiometer, Copenhagen, Denmark) for blood gas analysis, and the quantification of TCA cycle intermediates is detailed in the next subsection. The experimental protocol was concluded at T6 or before this point if the animal showed any signs of malignant hyperthermia. Animals were killed with pentobarbital + diphenylhydantoin 100 mg/kg in accordance with the American Veterinary Medical Association criteria. No animals were excluded.

### 2.4 Quantification of TCA cycle intermediates

At T0, T3 and T6, 10 mL of venous blood was sampled with a non‐heparinized syringe in vacutainers of 5 mL, which were immediately centrifuged for 10 min at 1560*g* in a prerefrigerated centrifuge at −20°C. The plasma was transferred to an Amicon 30 000 Da cut‐off filter (Millipore, Watford, UK), where centrifugation at 1560*g* for 15 min produced ultrafiltrate. The generated ultrafiltrate was stored at −60°C for analysis within 12 h. Previous studies have demonstrated the efficacy of this procedure for sample preparation and storage before quantification of TCA cycle intermediates (Forni et al., 2005; McKinnon et al., 2006).

The samples were transferred to the Chromatography Laboratory at Universidad Nacional de Colombia, where analysis of the samples was carried out using HPLC. Following the methodology described by Shurubor et al. (2016), the separation and quantification of the following acid patterns (>98% titration): pyruvic (millimolar), lactic (millimolar), succinic (micromolar), α‐ketoglutaric (micromolar), citric (micromolar), fumaric (micromolar) and malic (micromolar), was obtained. The method was validated for each of the intermediates at different dilutions, and for a mixture with all patterns, a determination coefficient (*R* ^2^) > 0.999 was present for all metabolites. The level of detection, level of quantification and *R* ^2^ of all analytes are depicted in Supporting Information, Table S1.

For all chromatographic separations, a Hitachi Primaide HPLC system (Hitachi High‐Technologies Corporation, Tokyo, Japan), equipped with a 1110 quaternary pump, 1210 autosampler and 1410 UV‒Vis detector were used. A Phenomenex C18 reverse phase column, Prodigy 5u ODS3 100A (250 mm × 4.6 mm) (Phenomenex, Torrance, CA, USA) was used. A 20 mM sodium phosphate buffer solution (pH 2.9) was used as the mobile phase. Standard and test samples were prepared in 10% perchloric acid.

The chromatographic analysis was carried out with a UV‒Vis detector adjusted to a wavelength of 210 nm and with a mobile phase flow of 0.45 mL/min in isocratic mode and varying the flow over time starting at 0.3 mL/min for 7 min, then at a flow of 0.45 mL/min for ≤18 min.

Blood concentrations were derived by correcting plasma concentrations (*C*_plasm_) for haematocrit according to the formula: $\mathrm{blood}\;\mathrm{concentration}=C_{\mathrm{plasm}}\times \left(1-\mathrm{haematocrit}\right).$

### 2.5 Statistical analysis

A sample size was not calculated since this is an exploratory analysis. Continuous variables were tested for normality using the Kolmogorov‒Smirnov test. Data were presented as the mean and SD for a normal distribution, or otherwise as medians and interquartile ranges (IQR).

To evaluate whether there were differences in terms of haemodynamic and metabolic variables between the endotoxin and control groups at T0, T3 and T6, a Mann‒Whitney *U*‐test was performed for independent populations, and an adjustment for continuity was made to calculate the *P*‐value under the standard normal distribution. To assess whether there were differences between medians within the same group at different time periods (T0, T3 and T6), we performed repeated‐measures ANOVA and consecutive Bonferroni correction at specific times when relevant *F*‐values were significant at the 5% level.

A linear regression model of the significantly elevated intermediates was performed to calculate the coefficient of determination between changes in their concentrations and changes in lactate concentrations and the L/P ratio. Finally, in addition to standard statistical analyses, *post hoc* effect size estimates using Cohen's *d* were performed to assess the magnitude of effect of the intervention on metabolic variables. The statistical analysis was performed using R software v.4.2.3 (R Foundation for Statistical Computing, Vienna, Austria).

## 3 RESULTS

The mean time to TS was 169 min, and the average dose of LPS needed for induction of endotoxic shock was 2.5 mg (Supporting Information, Table S2). Key haemodynamic variables and oxygen‐derived variables are shown in Supporting Information, Table S3. The medians of MAP and heart rate were significantly different between the endotoxin and control groups at T3 and T6. The global end‐diastolic volume was significantly lower at T6 in the endotoxin group than in the control group. In the endotoxin group, SVR showed a significant reduction between T0 and T3; afterwards, between T3 and T6, SVR increased, and stroke volume decreased. As expected, in control animals, no significant haemodynamic changes were observed. No differences in oxygen supply and oxygen consumption were observed between groups at any time.

**FIGURE 2:**
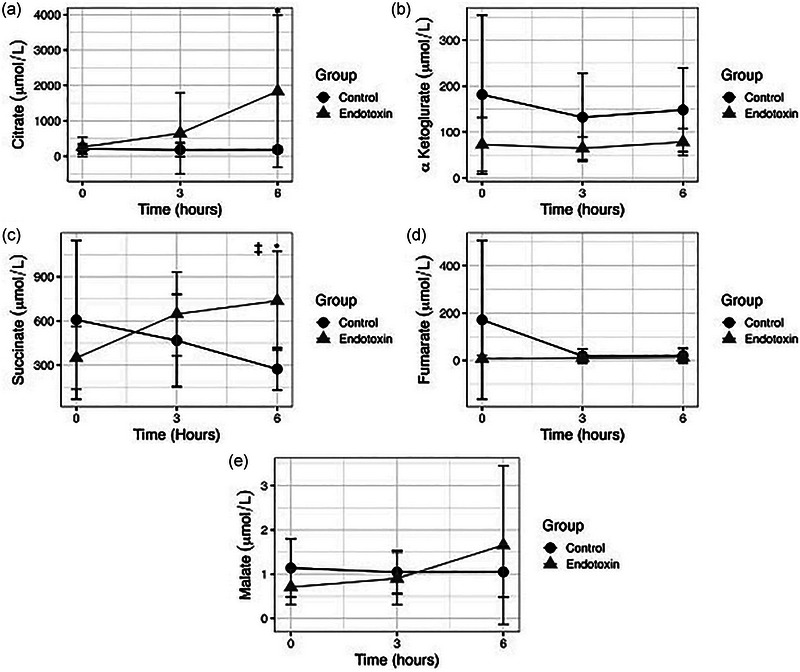
Time course of tricarboxylic acid cycle intermediates. Time course of citrate (a), α‐ketoglutarate (b), succinate (c), fumarate (d) and malate (e). Control group (*n* = 4); endotoxin group (*n* = 9). *P < 0.05 at T6 control versus endotoxin, ^†^ *P *< 0.05 T0 versus T6 in the endotoxin group.

The median plasma TCA cycle intermediates for both groups are detailed in Table 1 and Figure 2. Detailed concentrations of all HPLC analyses are described in Supporting Information, Tables S4 and S5. There was a significant increase at T6 for citrate and succinate in the endotoxin group compared with the control group: 453.1 (IQR 416–540) versus 190 (153.75–221.67) µmol/L (*P* = 0.03) for citrate and 700 (630–842) versus 241 (159.17–355.81) µmol/L (*P* = 0.03) for succinate. Plasma citrate concentrations in the endotoxin group increased between T0 and T6 [T0, 190 (156.67–313.33) µmol/L versus T6, 453,1 (416–540) µmol/L; *P* = 0.04; Figure 2a]. Succinate concentrations in the endotoxin group also increased between T0 and T6 [T0, 370 (303.33–406.67 µmol/L) versus T6, 700 (630–842) µmol/L; *P* = 0.01; Figure 2c]. No other TCA cycle intermediates in the endotoxin group showed any variations in plasma concentrations. Control animals showed stability of TCA cycle intermediates throughout all experimental protocols.

**TABLE 1:** Plasma tricarboxylic acid cycle intermediates, pyruvate, lactate and lactate:pyruvate ratio during the experimental period.

Parameter	Group	T0	T3	T6
Citrate, µM/L*	C	255.83 (163.75–296.67)	126.66 (38.33–271.66)	190.00 (153.75–221.66)
	E	190.00 (156.67–313.33)	290.00 (186.66–404,33)	453.1^†^ (416.00–540.00)
α‐Ketoglutarate, µM/L	C	133.33 (51.66–263.33)	125 (55.625–201.66)	128.33 (80–196.67)
	E	57.5 (40.00–83.33)	62.50 (45.67–80.00)	70.00 (63.33–110)
Succinate, µM/L*	C	523.5 (306.67–825)	414.58 (273.54–608.33)	241.65 (159.17–355.81)
	E	370.00 (303.33–406.67)	646.67 (482.5–693.33)	700.00^†^ (630–842.5)
Fumarate, µM/L	C	3.93 (3.46–4.66)	5.91 (3.37–21.58)	6.63 (4.49–22.9)
	E	3.73 (2.04–5.13)	4.97 (4.3–6.88)	7.03 (4.22–9.45)
Malate, µM/L	C	1.08 (0.6–1.63)	0.935 (0.69–1.29)	0.92 (0.63–1.35)
	E	0.81 (0.4–0.87)	0.95 (0.45–1.39)	0.82 (0.67–1.29)
Pyruvate, mM/L	C	0.94 (0.65–1.26)	0.97 (0.52–1.44)	0.97 (0.63–1.29)
	E	1.08 (0.88–1.29)	1.31 (0.94–1.46)	1.79 (1.24–1.94)
Lactate, mM/L^*^	C	2.68 (2.11–3.63)	1.98 (1.67–3.73)	1.9 (1.33–2.7)
	E	2.88 (2.51–3.49)	6.57 (5.15–7.31)	10.36 (6.56–15.7)^†‡^
Lactate:pyruvate ratio	C	3.19 (1.72 –5.8)	3.1 (1.17–7.58)	1.83 (1.54–3.24)
	E	2.56 (1.96–4.22)	5.57 (3.41–6.99)	4.14 (3.68–8.80)

*Note*: Values are represented as medians (interquartile range). Abbreviations: C, control group (*n* = 4); E, endotoxin group (*n* = 9). Differences between groups at each time point were obtained by the Mann‒Whitney *U*‐test, and differences in the same group at different times were obtained by repeated‐measures ANOVA with Bonferroni correction: *P < 0.05 at T6 control versus endotoxin, ^†^ *P *< 0.05 T0 versus T6 control versus endotoxin, ^‡^ *P *< 0.05 T3 versus T6 in the endotoxin group.

These differences were analysed after adjusting the data for haematocrit to derive blood concentrations of TCA intermediates. Detailed blood concentrations of TCA intermediates are reported in Supporting Information, Table S6. A significant increase in citrate and succinate blood concentrations was observed at T6 in the endotoxin group compared with the control group: 134.77 (107.81–151.65) versus 300 (226.66–376.97) µmol/L (*P* = 0.03) for citrate and 170.77 (108.67–252.62) versus 497 (434.70–615.02) µmol/L (*P* = 0.03) for succinate. Blood citrate concentrations in the endotoxin group increased significantly from T0 to T6 [T0, 122.4 (112.80–193.16) µmol/L versus T6, 300 (226.6–376.9) µmol/L; *P* < 0.05]. Likewise, blood succinate concentrations in the endotoxin group also increased significantly between T0 and T6 [T0, 269.5 (191.1–292.8) µmol/L versus T6, 497 (434.70–615.02) µmol/L; *P* = 0.01].

**FIGURE 3:**
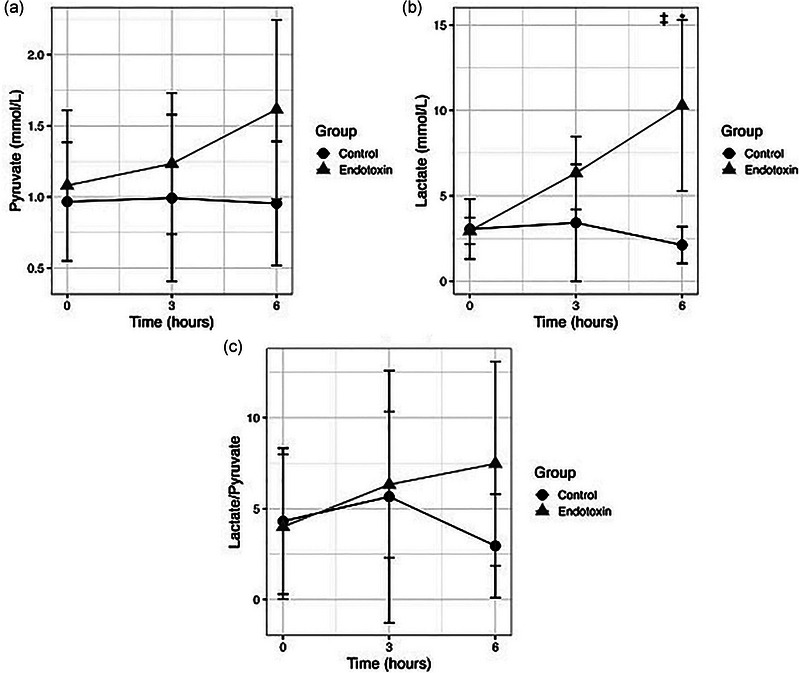
Time course of pyruvate, lactate and the lactate:pyruvate ratio. Time course of pyruvate (a), lactate (b) and the lactate:pyruvate ratio (c). *P < 0.05 at T6 control versus endotoxin; ^†^ *P *< 0.05 T0 versus T6 in the endotoxin group; ^‡^ *P *< 0.05 T3 versus T6 in the endotoxin group.

The median plasma lactate and pyruvate levels along with the L/P ratio are shown in Table 1 and Figure 3. A significant increase in lactate concentration at T6 was observed in the endotoxin group compared with the control group [10.36 (IQR 6.56–15.73) versus 1.90 (1.33–2.7) mmol/L; *P* = 0.0001]. Plasma lactate in the endotoxin group increased between T0 and T6 [T0, 2.88 (2.51–3.49 mmol/L) versus T6, 10.36 (6.56–15.73 mmol/L); *P *= 0.0001] and between T3 and T6 [T3, 6.57 (5.15–7.31) mmol/L versus T6, 10.36 (6.56–15.73) mmol/L; *P* = 0.04; Figure 3b]. The median L/P ratio did not show significant differences in the endotoxin group compared with the control group at any point of the experiment, nor during the course of endotoxaemia in the endotoxin group (Figure 3c). Lactate, pyruvate and the L/P ratio in control animals did not show any changes during the experimental protocol.

**FIGURE 4:**
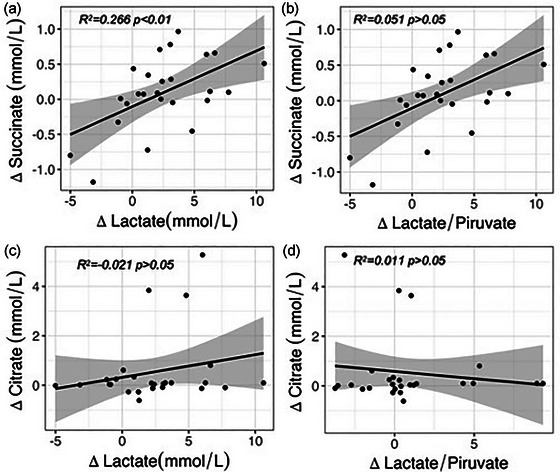
Linear regression analysis of changes (∆) in succinate and citrate for determination of changes (∆) in lactate and the lactate:pyruvate ratio. Linear regressions of ∆succinate–∆lactate (a); ∆succinate–∆lactate:pyruvate ratio (b); ∆citrate–∆lactate (c); and ∆citrate–∆lactate:pyruvate ratio (d). *R* ^2^ was obtained by the least squares regression analysis method.

Linear regressions of the elevated TCA intermediates (succinate and citrate) for determination of changes in lactate concentrations and the L/P ratio are depicted in Figure 4. Changes in succinate concentrations were associated with variations in lactate concentrations (*R* ^2 ^= 0.266, *P* = 0.004; Figure 4a) but not with the L/P ratio (*R* ^2 ^= 0.051, *P* = 0.142; Figure 4b). Changes in citrate concentrations were not related to variations in lactate concentrations (*R* ^2 ^= −0.021, *P* = 0.264; Figure 4c) or to the L/P ratio (*R* ^2 ^= 0.012, *P* = 0.499; Figure 4d).

### 3.1 Effect size analysis in TCA cycle intermediates: a *post hoc* power analysis

The comparison of citrate levels between the control and intervention groups at T6 revealed a large positive effect size (*d* = 1.24, 95% confidence interval: −0.19 to 2.66), suggesting a substantial increase in citrate levels in the intervention group, although the confidence interval indicates some uncertainty about the precise magnitude of this effect. Likewise, the analysis of succinate levels at T6 showed a large positive effect size (*d* = 1.57, 95% confidence interval: 0.09–3.06), indicating a significant increase in succinate levels with a relatively high degree of certainty regarding the magnitude of the effect. In contrast, the comparison of citrate levels between T0 and T6 demonstrated a medium positive effect size (*d* = 0.71, 95% confidence interval: −1.74 to 0.32), indicating a moderate increase in citrate levels over time, although with some uncertainty regarding the direction and magnitude of the effect. Meanwhile, the comparison of succinate levels over the same time period revealed a large positive effect size (*d* = 1.38, 95% confidence interval: 2.50–0.27), suggesting a significant increase in succinate levels, with strong confidence in the significance of this effect.

## 4 DISCUSSION

In this animal model of endotoxic shock, the haemodynamic response was characterized by an initial decrease in SVR, MAP and global end‐diastolic volume. Later, a decrease in stroke volume was observed after restoration of SVR with the use of noradrenaline. During this resuscitated endotoxic shock, systemic oxygen supply and oxygen consumption remained constant throughout the experiment. With regard to the metabolic response, the endotoxic group showed a significant increase in succinate, citrate and lactate concentrations. The fact that the median plasma L/P ratio remained <10 in the endotoxin group and the absence of significant variations through time in this group depicts global aerobic conditions with a catalytic predominance, because this metabolic response mainly reflects a state of activation of intermediary metabolism.

Plasma concentrations and analysis of these TCA cycle intermediates during endotoxic shock have not been described previously. Our results are in line with animal data reporting increased concentrations of TCA cycle metabolites (citrate, fumarate and malate) in liver biopsies in a mouse model of sepsis induced by caecal ligation and perforation (Whelan et al., 2014). However, other animal studies have reported no changes in TCA cycle metabolites in cardiac tissue from a murine caecal ligation and perforation model (Hotchkiss et al., 1991) and from a swine endotoxic shock model (Lado‐Abeal et al., 2018), with both these investigations collecting samples >24 h after shock. Furthermore, some data in mice report decreased concentrations of fumarate and malate in kidney tissue obtained 8 h after shock (Waltz et al., 2016), and more recently, a model of chronic LPS exposure reported decreased concentrations in plasma of TCA intermediates (Hernandez‐Baixauli et al., 2023). In all the cited studies, TCA cycle intermediates were measured at different stages of sepsis and in different tissues; therefore, plasma concentrations of TCA intermediates ultimately represent the time‐dependent contribution of multiple tissues with different metabolic responses. Our results represent a global perspective, in which a predominant catalytic profile has developed during the early phase of endotoxaemia.

Our results are also consistent with metabolomic data from septic patients, which report elevated plasma citrate concentrations in non‐surviving subjects (Langley et al., 2014; Liu et al., 2016) and in patients with deteriorating organic function (Cambiaghi et al., 2017). Concerning increased succinate levels, their increase has been reported in a retrospective cohort of septic shock patients (Mickiewicz et al., 2014) and in various animal shock models (D'Alessandro et al., 2017; LaCroix et al., 2023; Reisz et al., 2018). Most of these studies associate the increase in citrate and succinate with a poor prognosis, because their elevation denotes a failure of the TCA cycle in the uptake of these metabolites, which represents a disturbance in the redox state of the cell (Cerra et al., 1990; Owen et al., 2012). However, in our model, the observed increase in these TCA cycle intermediates took place in aerobic conditions, which is consistent with the low L/P ratio and stable oxygen supply during endotoxic shock. In these circumstances, fluctuations in plasma succinate were the only metabolic variable associated with changes in aerobic lactate production (Figure 4a). Consequently, it can be inferred that in these experimental conditions, the variations in lactate concentrations were predicted by the changes in succinate production. Moreover, the increases in plasma succinate were not associated with changes in the L/P ratio, which is expected because an increase in the L/P ratio is mainly a reflection of the induction of anaerobiosis. Therefore, in our study succinate concentrations were associated with the amount of aerobic lactate production.

In humans, succinate is the product of 31 enzymatic reactions, of which canonical TCA cycle activity is the main source in aerobic conditions (Chinopoulos, 2019). Our results showed a marked increase in succinate after 6 h of endotoxin administration, with succinate being the only intermediate related to the changes in aerobic lactate production. This suggests that succinate production during endotoxaemia is independent of canonical TCA cycle activity, because when canonical functioning takes place, the plasma concentrations of TCA intermediates decrease (Beloborodova et al., 2019; Forni et al., 2005; Langley et al., 2014; Liu et al., 2016). Therefore, there are three main mechanisms that could explain the increase in succinate in our model: the first is related to the GABA shunt; this pathway produces succinate from α‐ketoglutarate, bypassing the TCA cycle, and its increase has been documented in macrophages during pro‐inflammatory activation (Liu et al., 2021; Tannahill et al., 2013). The second mechanism is mediated by the inhibition of succinate dehydrogenase or respiratory complex II. Succinate dehydrogenase is a TCA cycle enzyme responsible for the oxidation of succinate to fumarate (reduction of FAD to FADH_2_); its inhibition leads to succinate accumulation and has been associated with the production of bacterial‐derived phenolic acids (Beloborodova et al., 2019; Fedotcheva et al., 2018) and itaconic acid, a byproduct of the TCA cycle that shows increased levels during pro‐inflammatory signalling (Cordes et al., 2016; Lampropoulou et al., 2016). The third mechanism is performed by the reverse function of the TCA cycle leading to the production of succinate from oxaloacetate; this non‐canonical TCA cycle activity has been described only in hypoxic conditions (Chinopoulos, 2013, 2019; Chouchani et al., 2014). Given that our model did not alter systemic oxygen supply, the increase in plasma succinate concentrations could be explained by a combination of the first two mechanisms, suggesting a potential role for succinate as a biomarker of aerobic lactate production.

Identifying the variation in TCA cycle intermediates in a septic model allows an in‐depth understanding of one of several mechanisms underpinning the dynamic metabolic response to sepsis. Recently, a large body of evidence has accumulated regarding the role of metabolism in the regulation of the immune system and adaptation to hypoxia (Choi et al., 2021; Harber et al., 2020; Maurice & Sadikot, 2023; Wasyluk & Zwolak, 2021). Succinate is a particularly important intermediate, because it increases pro‐inflammatory signalling (Liu et al., 2021; Ryan & O'Neill, 2017, 2020), and its levels regulate hypoxia response pathways by stabilizing hypoxia‐induced factor‐1α (Koivunen et al., 2008; Selak et al., 2005), increasing production of reactive oxygen species and regulating mitochondrial fission (Chouchani et al., 2014; Hernandez‐Baixauli et al., 2023; Lu et al., 2018); phenomena of particular relevance to the host response during sepsis. The results of the present work suggest that during the early stages of endotoxic shock, succinate levels predict the increase in lactate production in aerobic conditions; in this phase, hyperlactataemia reflects a hypercatabolic response, in which the increase in succinate levels might play a role in immunoregulation and metabolic homeostasis, even without the development of anaerobic metabolism; further studies are needed to address this hypothesis and to confirm that abnormal TCA cycle function in the early phases of sepsis is a product of inflammatory signalling and worsens bioenergetics and immune function during sepsis.

The present study has some limitations. Firstly, the model of endotoxic shock did not reproduce the clinical conditions of a septic patient, because septic shock is normally not mediated only by LPS signalling and usually involves a wide variety of pathogens and damage‐associated molecular patterns, hence the results of this translational research need to be confirmed in the clinical arena. Secondly, we did not measure TCA intermediates in tissue samples, hence the particular contributions of different tissues, such as liver, kidney and heart, were beyond the scope of this work; Thirdly, we did not measure all TCA cycle intermediates because some of them are very unstable in plasma; for example, oxaloacetate has a plasma half‐life of almost 70 s, which makes its analysis very difficult (McKinnon et al., 2006). Fourthly, the administration of vasopressors could lead to an increase in metabolism and aerobic lactate production. However, our protocol only included the use of noradrenaline at doses that are not associated with hyperlactataemia, as has been shown in a landmark clinical trial in septic shock (Myburgh et al., 2008). Finally, our results represent the kinetics of TCA intermediates in the early phase of endotoxaemia; therefore, in more severe scenarios with predominant tissue hypoperfusion, the plasma concentrations of TCA intermediates might differ from our results.

## 5 CONCLUSION

During the early phase of this model of endotoxic shock, citrate and succinate were significantly elevated. Of all the TCA cycle intermediates measured, only the increase in succinate concentrations was associated with the increase in lactate in global aerobic conditions, highlighting the presence of a hypercatabolic response, in which aerobic production of lactate was determined by the change in succinate concentrations during this early phase of sepsis.

## AUTHOR CONTRIBUTIONS

Experiments were performed in the Instituto de Simulación Médica (INSIMED) simulation laboratory, Bogota DC. Juan Daniel Caicedo, Jorge Ivan Alvarado, Juan Jose Diaztagle, Candida Rosa Diaz and Luis Eduardo Cruz contributed to the conception and design of research and drafting of the work. Juan Daniel Caicedo and Jorge Ivan Alvarado performed experiments and analysed the data. All authors have read and approved the final version of this manuscript and agree to be accountable for all aspects of the work in ensuring that questions related to the accuracy or integrity of any part of the work are appropriately investigated and resolved. All persons designated as authors qualify for authorship, and all those who qualify for authorship are listed.

## CONFLICT OF INTEREST

None declared.

## FUNDING INFORMATION

None.

## Supporting information




**Supplemental digital content 1**: Arrive 2.0 Checklist for authors.

**Supplemental digital content 2**:
**Table S1**. Levels of detection (LOD), levels of quantification (LOQ) and determination coefficients (*R* ^2^) of HPLC‐quantified analytes after the calibration process.
**Table S2**. General characteristics of animals in the control (C) and endotoxin (E) groups.
**Table S3**. Haemodynamic variables, O_2_‐derived parameters during the experimental period.
**Table S4**. Individual HPLC analysis data for plasma TCA cycle intermediates.
**Table S5**. Individual HPLC analysis data for plasma lactate, pyruvate and L/P ratio.
**Table S6**. Individual HPLC analysis data for whole blood TCA cycle intermediates.

## Data Availability

The supplementary material, analysis codes and raw data are available from a GitHub repository: https://github.com/candidadiaz/krebscycle.git
