# Supplementary material for: Increase in plasma succinate is associated with aerobic lactate production in a model of endotoxic shock
Source: Exp Physiol. 2025 Mar 19;110(4):550–60. doi: 10.1113/EP092109 (PMC11963902; doi:10.1113/EP092109)
Supplement: Supplementary file 2 — Supplemental digital content 2: Table S1. Levels of detection (LOD), levels of quantification (LOQ) and determination coefficients (R 2) of HPLC‐quantified analytes after the calibration process. Table S2. General characteristics of animals in the control (C) and endotoxin (E) groups. Table S3. Haemodynamic variables, O2‐derived parameters during the experimental period. Table S4. Individual HPLC analysis data for plasma TCA cycle intermediates. Table S5. Individual HPLC analysis data for plasma lactate, pyruvate and L/P ratio. Table S6. Individual HPLC analysis data for whole blood TCA cycle intermediates. [file EPH-110-550-s001.pdf]

Supplementary Tables.

Table S1. Levels of detection (LOD), levels of quantification (LOQ), and determination coefficients (R^2) of HPLC quantified analytes after the calibration process.

ANALYTES	LOD	LOQ	R2
**PYRUVIC ACID**	0,000176	0,000535	1
**MALIC ACID**	0,00156	0,00474	0,9995
**LACTIC ACID**	0,003	0,008	0,9991
**α-KETOGLUTARIC ACID**	1,329E-04	0,000402	0,9989
**CITRIC ACID**	1,009E-03	0,00306	0,9997
**FUMARIC ACID**	1,478E-05	0,0000447	1
**SUCCINIC ACID**	0,00287	0,00870	1

Table S2. General characteristics of animals in control (C) and endotoxin (E) groups.

Animal No	C1	C2	C3	E1	E2	E3	E4	E5	E6	E7	E8	E9
**Sex**	F	F	F	F	F	F	F	F	F	F	F	F
**Weight (Kg)**	48	50	48	50	52	52	49	48	47	50	50	50
**Corporal surface area (m^2)**	0.93	0.96	0.93	0.96	0.98	0.98	0.94	0.93	0.92	0.96	0.96	0.96
**Age (months)**	5	4	4	5	5	5	4.5	5	4	5	4	4
**Time to shock (min)**				96	129	95	160	270	82	133	360	200
**LPS dose (µg)**				1429	2052	1469	2435	4128	1126	2039	5000	3145
**Median volume of fluid administration previous to T0 (mL).**	964	970	1320	1466	1480	1485	1467	1467	1461	1670	1470	1470
**Median volume of fluid administration from T0 to T6 (mL).**	2472	2560	2472	2560	2648	2648	2516	2472	2428	2560	2560	2560

Table S3. Hemodynamic variables, O_2-derived parameters during the experimental period.

		T0	T3	T6
**HR** (bpm) *†	C	109 (103-111)	106 (86-122)	103 (75-128)
	E	99 (92-103)	141 (97-165)	159 (119-177) ¥
**MAP** (mmHg) *†	C	80 (71-81)	85 (63-92)	75 (73-81)
	E	75 (68-82)	52 (50-59) ‡	55 (44-64) ¥
**CVP** (mmHg)	C	11.6 (7.1-13.3)	12.6 (10.5-13)	10.3 (8-11.3)
	E	11.3 (9.7-13.5)	11.9 (9-12.6)	8.7 (7.4-9.5)
**SVR** (dyn/cm^5/m^2)	C	1297 (1083-1339)	1123 (850-1523)	1016 (1013-1503)
	E	1117 (786-1165)	776 (487-960) ‡	967 (605-1248) §
**CO** (L/min)	C	4.89 (3.24-5.41)	4.34 (4.15-4.99)	5.89 (4.09-6.23)
	E	4.12 (4.05-5.58)	4.3 (4.01-7.66)	5.25 (2.82-7.16)
**SV** (ml)	C	49 (30-51)	47 (36-47)	52 (50-57)
	E	51 (43-55)	40 (33-63)	34 (18-44) ¥
**GEDV** (ml) †	C	556 (474-591)	592 (458-608)	630 (550-636)
	E	504 (467-641)	455 (430-485)	445 (419-452) ¥
**DO_2** (mL/min/kg)	C	13.82 (6.49-15.18)	11.45 (10.60-12.46)	14.73 (10.03-16.78)
	E	14.48 (11.99-15.46)	14.16 (10.96-17.87)	15.43 (7.79-19.97)
**VO_2** (mL/min/kg)	C	2.07 (1.86-2.44)	2.35 (1.45-2.37)	2.17 (1.59-3.24)
	E	1.88 (1.83-3.56)	2.23 (1.3-3.29)	2.25 (1.64-2.96)
**O_2ER** (%)	C	19.87 (13.99-28.18)	18.11 (11.96-22.09)	17.14 (11.06-21.34)
	E	16.2 (11.32-23.89)	13.63 (8.94.24.46)	23.20 (11.75-34.99)

Values are represented as medians (interquartile range – IQR). C denotes the control group (n=4); E denotes the endotoxin group (n=9). Differences between groups at each time point were obtained by the Mann–Whitney U test, and differences in the same group at different times were obtained by repeated-measures analysis of variance with Bonferroni correction: * $p<0.05$ at T3 control vs endotoxin, † $p<0.05$ at T6 control vs endotoxin, ‡ $p<0.05$ T0 vs T3 in endotoxin group, ¥ $p<0.05$ T0 vs T6 in the endotoxin group, § $p<0.05$ T3 vs T6 in the endotoxin group. HR: Heart rate; MAP: mean arterial pressure; CVP: central venous pressure; CO: cardiac output; SV: stroke volume; GEDV: global end diastolic volume; PPV: pulse pressure variation; DO_2: oxygen delivery; VO_2: oxygen consumption; O_2ER: oxygen extraction rate

Table S4. Individual data of plasma tricarboxylic acid cycle intermediates obtained by HPLC analysis.

	CITRATE µM/L			KETOGLUTARATE µM/L			SUCCINATE µM/L			FUMARATE µM/L			MALATE µM/L		
**Animal Nº**	**T0**	**T3**	**T6**	**T0**	**T3**	**T6**	**T0**	**T3**	**T6**	**T0**	**T3**	**T6**	**T0**	**T3**	**T6**
Endotoxin 1	316,67	403,33	500	83,33	103,33	70	76,67	786,67	1296,67	2,1	4,97	4,22	1,36	1,39	5,54
Endotoxin 2	190	290	303,33	30	50	53,33	303,33	1270	90	5,13	4,3	1,7	0,27	0,29	0,41
Endotoxin 3	230	106,67	913,33	40	43,33	110	400	403,33	1063,33	3,04	2,3	65,9	0,25	0,45	0,52
Endotoxin 4	10	110	453.1	40	30	33,33	370	482,5	582,5	5,27	10,47	9,45	0,43	0,03	0,74
Endotoxin 5	156,67	500	540	57,5	62,5	70	513,33	586,67	842,5	3,73	6,17	4,1	0,87	0,55	0,67
Endotoxin 6	313,33	186,67	546,33	40	80	110	7,23	646,67	630	3,67	52,5	12,67	0,4	1,34	3,85
Endotoxin 7	46,67	3686,67	430	220	46,67	63,33	716,67	263,33	700	2,8	4,4	7,43	0,81	0,95	0,82
Endotoxin 8	933,33	320	416	63,33	86,67	120	350	693,33	783,33	42,97	6,89	7,03	1,14	1,62	1,29
Endotoxin 9	170	233,33	333,33	83,33	80	76,67	406,67	693,33	646,67	4,93	4,2	4,43	0,83	1,51	1,07
MEDIAN	*190*	*290*	*453,1*	*57,5*	*62,5*	*70*	*370*	*646,67*	*700*	*3,73*	*4,97*	*7,03*	*0,81*	*0,95*	*0,82*
RIQ1	*156,67*	*186,66*	*416*	*40*	*46,67*	*63,33*	*303,33*	*482,5*	*630*	*2,04*	*4,3*	*4,22*	*0,4*	*0,45*	*0,67*
RIQ3	*313,33*	*403,33*	*540*	*83,33*	*80*	*110*	*406,67*	*693,33*	*842,5*	*5,13*	*6,88*	*9,45*	*0,87*	*1,39*	*1,29*
Control 1	10	40	75	53,33	42,5	70	607	312,5	323,3	2,85	0,79	3,18	1,56	1,16	1,2
Control 2	215	446,67	180	46,67	60	83,33	1210	883,33	160	6,07	7,6	4,93	0,55	0,71	0,56
Control 3	296,67	33,33	286,67	413,33	190	173,33	173,33	156,6	156,67	3,67	4,23	8,33	1,84	1,69	1,79
Control 4	296,67	213,33	200	213,33	236,67	266,67	440	516,67	453,33	4,19	63,53	66,6	0,61	0,63	0,65
MEDIAN	*255,835*	*126,665*	*190*	*133,33*	*125*	*128,33*	*523,5*	*414,585*	*241,65*	*3,93*	*5,915*	*6,63*	*1,085*	*0,935*	*0,925*
RIQ1	163,75	38,33	153,75	51,66	55,62	80	306,67	273,54	159,17	3,46	3,37	4,49	0,6	0,69	0,63
RIQ3	296,67	271,67	221,67	263,33	201,66	196,67	825	608,33	355,81	4,66	21,58	22,9	1,63	1,29	1,35

Table S5. Individual data of plasma lactate, pyruvate and L/P ratio obtained by HPLC analysis.

Animal Nº	PYRUVATE mM/L			LACTATE mM/L			L/P RATIO		
	T0	T3	T6	T0	T3	T6	T0	T3	T6
Endotoxin 1	1,45	1,5	1,24	3,49	5,7	16,32	2,41	3,79	13,12
Endotoxin 2	1,07	0,94	0,99	2,88	6,57	3,4	2,69	6,99	3,43
Endotoxin 3	0,2	0,36	0,6	2,85	5,15	11,78	14,27	14,3	19,63
Endotoxin 4	1,08	0,77	2,25	2,11	8,36	16,11	1,96	10,9	7,16
Endotoxin 5	1,14	1,25	1,35	1,66	2,56	4,95	1,45	2,05	3,68
Endotoxin 6	0,88	1,42	1,79	3,73	9,7	15,73	4,22	6,83	8,8
Endotoxin 7	0,55	1,31	1,79	2,51	7,31	7,39	4,53	5,57	4,14
Endotoxin 8	1,29	1,46	1,94	3,29	4,56	6,56	2,56	3,12	3,38
Endotoxin 9	2,06	2,09	2,6	4,02	7,12	10,36	1,95	3,41	3,99
MEDIAN	*1,08*	*1,31*	*1,79*	*2,88*	*6,57*	*10,36*	*2,56*	*5,57*	*4,14*
RIQ1	*0,88*	*0,94*	*1,24*	*2,51*	*5,15*	*6,56*	*1,96*	*3,41*	*3,68*
RIQ3	*1,29*	*1,46*	*1,94*	*3,49*	*7,31*	*15,73*	*4,22*	*6,99*	*8,80*

Control 1	0,68	0,44	0,68	3,01	2,13	1,17	4,43	4,88	1,72
Control 2	1,21	1,6	1,25	2,36	1,2	2,42	1,95	0,75	1,93
Control 3	0,55	0,54	0,49	5,49	8,53	3,52	9,93	15,71	7,18
Control 4	1,43	1,39	1,39	1,36	1,83	1,38	0,95	1,32	0,99
MEDIAN	*0,94*	*0,97*	*0,97*	*2,68*	*1,98*	*1,9*	*3,19*	*3,1*	*1,83*
RIQ1	0,65	0,52	0,63	2,11	1,67	1,33	1,72	1,17	1,54
RIQ3	1,26	1,44	1,29	3,63	3,73	2,7	5,8	7,58	3,24

Table S6. Individual data of tricarboxylic acid cycle intermediates adjusted by hematocrit to derive blood concentrations.

	CITRATE µM/L			KETOGLUTARATE µM/L			SUCCINATE µM/L			FUMARATE µM/L			MALATE µM/L		
**Animal Nº**	**T0**	**T3**	**T6**	**T0**	**T3**	**T6**	**T0**	**T3**	**T6**	**T0**	**T3**	**T6**	**T0**	**T3**	**T6**
Endotoxin 1	193,17	258,13	300,00	50,83	66,13	42,00	46,77	503,47	778,00	1,28	3,18	2,53	0,83	0,89	3,32
Endotoxin 2	119,70	208,80	209,30	18,90	36,00	36,80	191,10	914,40	62,10	3,23	3,10	1,17	0,17	0,21	0,28
Endotoxin 3	156,40	73,60	602,80	27,20	29,90	72,60	272,00	278,30	701,80	2,07	1,59	43,49	0,17	0,31	0,34
Endotoxin 4	6,90	74,80	10,67	27,60	20,40	21,33	255,30	328,10	372,80	3,63	7,12	6,05	0,30	0,02	0,47
Endotoxin 5	112,80	365,00	394,20	41,40	45,63	51,10	369,60	428,27	615,03	2,68	4,50	2,99	0,63	0,40	0,49
Endotoxin 6	225,60	130,67	376,97	28,80	56,00	75,90	5,21	452,67	434,70	2,64	36,75	8,74	0,29	0,94	2,65
Endotoxin 7	33,13	2543,80	305,30	156,20	32,20	44,97	508,83	181,70	497,00	1,99	3,04	5,28	0,58	0,66	0,58
Endotoxin 8	718,67	227,20	287,04	48,77	61,53	82,80	269,50	492,27	540,50	33,08	4,89	4,85	0,88	1,15	0,89
Endotoxin 9	122,40	158,67	226,67	60,00	54,40	52,13	292,80	471,47	439,73	3,55	2,86	3,01	0,60	1,03	0,73
MEDIAN	122,40	208,80	300,0*†	41,40	45,63	51,10	269,50	452,67	497,0*†	2,68	3,18	4,85	0,58	0,66	0,58
RIQ1	72,97	102,73	217,98	27,40	31,05	39,40	118,93	303,20	403,75	2,03	2,95	2,76	0,23	0,26	0,41
RIQ3	209,38	311,57	385,58	55,42	58,77	74,25	331,20	497,87	658,41	3,59	6,00	7,39	0,73	0,98	1,77
Control 1	5,90	27,20	53,25	31,47	28,90	49,70	358,13	212,50	229,54	1,68	0,54	2,25	0,92	0,79	0,85
Control 2	154,80	299,27	126,00	33,60	40,20	58,33	871,20	591,83	112,00	4,37	5,09	3,45	0,40	0,48	0,39
Control 3	222,50	23,00	180,60	310,0	131,10	109,20	130,00	108,05	98,70	2,75	2,92	5,25	1,38	1,16	1,13
Control 4	234,37	149,33	142,00	168,5	165,67	189,33	347,60	361,67	321,87	3,31	44,47	47,29	0,48	0,44	0,46
MEDIAN	188,65	88,27	134,00	101,0	85,65	83,77	352,87	287,08	170,77	3,03	4,01	4,35	0,70	0,63	0,66
RIQ1	43,13	24,05	71,44	32,00	31,73	51,86	184,40	134,17	102,03	1,95	1,13	2,55	0,42	0,45	0,41
RIQ3	231,40	261,78	170,95	274,6	157,03	169,30	742,93	534,29	298,79	4,10	34,63	36,78	1,26	1,07	1,06

Differences between groups at each time point were obtained by the Mann–Whitney U test, and differences in the same group at different times were obtained by repeated-measures analysis of variance (ANOVA) with Bonferroni correction: * $p<0.05$ at T6 control vs endotoxin, † $p<0.05$ T0 vs T6 control vs endotoxin.
